# Risk factors associated with diabetes in periurban community, Lahore Pakistan

**DOI:** 10.12669/pjms.336.13323

**Published:** 2017

**Authors:** Hina Ahmed, Inayat Thaver, Iram Manzoor

**Affiliations:** 1 Dr. Hina Ahmed, MBBS, PGD Nutrition, M-Phil Community Medicine. Assistant Professor, Department of Community Health Sciences, FMH College of Medicine & Dentistry, ahore, Pakistan; 2 Dr. Inayat Thaver, Health & Population Advisor, Mustashaar, Social Development Advisors. Department of Community Health Sciences, FMH College of Medicine & Dentistry, ahore, Pakistan; 3 Prof. Dr. Iram Manzoor, MBBS, FCPS Community Medicine. Department of Community Health Sciences, FMH College of Medicine & Dentistry, ahore, Pakistan

**Keywords:** Diabetes, Obesity, Positive family history

## Abstract

**Objectives:**

To identify factors (sociodemographic characteristic, behavioural factors, health care advice and physical measurements like weight, height, waist and hip circumferences) associated with diabetes.

**Methods:**

This study was conducted in the Nain Sukh which is a peri-urban area near Lahore between January till August 2016. A sum of 1080 households of both gender with age between 15-69 years were interviewed through a structured questionnaire and necessary measurements were taken. The data analysis was done by using SPSS version 17. All the recommended ethical clearance both institutional as well as individual levels were duly taken.

**Result:**

The mean age of the participants was 34 years ± 14SD with female predominance. Total prevalence of diabetes was found to be 9.8% out of which 83% were females between age of 45-69 years (p<0.00). Diabetes was significantly associated with obesity with 33% participants were overweight while 42% were obese (p<0.00). Diabetes was also significantly associated with central obesity, positive family history (p<0.000). Almost 45% of the households were advised to reduce weight and take special diet (p<0.000).

**Conclusion:**

The diabetes is significantly associated with positive family history and deranged BMI both overweight and obesity along with central obesity. This can only be prevented by health education and life style modifications.

## INTRODUCTION

Pakistan is the sixth most populous country of the world.^1^ Since its inception, Pakistan is facing many social and health related problem. Non communicable diseases are posing great threat to the developing country like Pakistan.^1^ Among these diabetes is posing a great threat. National diabetic survey of Pakistan by Diabetes Association of Pakistan (DAP)and WHO showed that the overall prevalence of diabetes as 11.5% and by the year 2035, the figures would be up to 12.8 million and Pakistan would be categorized as 8^th^ among the world’s top 10 countries having increased prevalence of diabetes.^2^,^3^

Many comorbid conditions are associated with Diabetes It is the complex disease which leads to metabolic derangement.^4^ Many factors like obesity, dyslipidemia, and sedentary life style, smoking and family history have been found to be associated with diabetes.^5^ Obesity is considered as one of the leading cause of insulin resistance due to chronic low grade inflammation in adipose tissue.^6^ Obesity and overweight can be assessed by different anthropometric methods but most frequently used in many epidemiological studies are through the determination of Body Mass Index (BMI) and Waist- Hip Ratio. Although it is not the gold standard for the measurement of fat distribution in the body but is the most convenient one to be employed in clinical practice.

Traditionally, studies on diabetes do focus on rural and urban differences. However with rapid urbanization and its consequences in Pakistan, it is also prudent to understand not only the magnitude but also the factors associated with diabetes. The presents study had been conducted so as to determine the relationship of different sociodemographic factors (age, sex, education and marital status), behavioral pattern and health seeking appraisal and anthropometric measures like Body Mass Index (BMI) and Waist Hip Ratio (WHR) with diabetes in periurban community of Lahore. This community based study highlights the magnitude as well associated factors which also need to be considered while making urban health plans and outreach services. Among others it illustrates that in spite of traditional beliefs, obesity is also prevalent in peri-urban areas.

The objective of this study was to identify factors (sociodemographic characteristic, behavioral factors, health care advice and physical measurements like weight, height, waist and hip circumferences) associated with diabetes.

## METHODS

This study was conducted in the Nain Sukh which is a peri-urban area near Lahore. Nain Sukh comes under union council with an estimated population of 35,000-40,000. An identified extended locality was selected and data was collected from all the households having a person aged between 15-69 years willing to participate in the study and who had been living in this area for at least six months. Therefore a sum of 1080 households got included having both males and females through non probability technique. A desired quota was determined by using Random Walk Quota sampling technique.^1^,^4^

The survey data collection was done by interviews of the household fulfilling the inclusion criteria and necessary measurements were determined. The questionnaire consisted of information regarding the sociodemographic profile, smoking habits, their appraisal regarding their health, family history of diabetes through a structured questionnaire. The weight, height, Waist and Hip circumferences were measured by following the WHO protocol for the calculation of body Mass Index(BMI) and Waist Hip ratio(WHR).^5^ The random blood sugar was measured by using pre-calibrated portable glucometer(Accu-Check) under aseptic measures. The cut off for the determination of random blood sugar was followed under the guide line of American Diabetic Association whereby all participants with blood sugar random ≤140 mg/dl were declared as having normal blood sugar levels while households with random blood sugar levels more than or equal to 200 mg/dl were declared as diabetics.^1^,^5^

Once the data was collected data cleaning was done for the missing data and data entry was done by using SPSS version 17. All the recommended ethical clearance both institutional as well as individual levels were duly taken.

## RESULTS

The results are based on the data from 1080 respondents. The mean age of the participants was 34 years ±14SD; median 30 years and mode 50 years. Most of the participants interviewed were female 871(80.6%) and 7.2% of the participants had an education from Madrasah/Quran (Table-I). Most of the participants were not gainfully employed (85.6%) with family income less than rupees 25,000/month. Interestingly 74%of the participants owned the houses in which they were living. The total number of participant who were smokers were 9.7%. The socio-demographic characteristics of the households is shown in Table-I.

**Table-I:** Sociodemographic Profile of the participants.

*Sociodemographic Profile of the participants N=1080*		*n*	*Percentage(%)*
Age in years (mean)		33.5	
Gender	Male	209	19.4
	Female	871	80.6
Education	Quran/Madrasah	939	86.9
	Up to primary	141	13.1
Marital status	Married	736	68
	Unmarried	307	28.4
	Widowed	30	2.8
	Separated	1	0.1
	Divorced	6	0.6
Working status	Un Employed	815	75.5
	Government Job	78	7.2
	Non-Government job	187	17.3

Total number of participants who had diabetes based on the history during past 12 months were 9.8%. Majority (83%) of the participant who had diabetes were females (Table-II). As expected, 65% of the diabetic had the age between 45-69 years (p=<0.00) (Table-II). Moreover, diabetes had been found to significantly associated with overweight and obesity (Table-III). Among the diabetic participants, 33% were overweight while 42% were obese (p<0.000). The central obesity was also significantly associated with Diabetes (Table-III). Almost half (43%) of the diabetic had positive family history of diabetes (p,0.000) (Table-IV). Nearly 45% of the households were advised to reduce weight (p<0.000) (Table-IV). Furthermore, more than half of the participants were asked to have special diet for diabetes (p<0.000).

**Table-II:** Sociodemographic profile and its relationship with diabetes.

*Sociodemographic profile and its relationship with diabetes among the households*						

*Variables*		*Diabetes n=106(%)*	*No diabetes n =974(%)*	*X^2^*	*df*	*p-value*
Age	15-29	15(14)	502(51)	95	2	0.000
	30-44	22(21)	255(23)			
	45-69	69(65)	217(22)			
Gender	Male	18(17)	191(20)	0.423	1	0.307
	Female	88(83)	783(80)			
Marital status	Married	90(85)	646(66)	44	4	0.000
	Unmarried	7(7)	300(3.3)			
	Separated	1(1)	0			
	Divorced	0	6(0.6)			
	Widowed	0	22(2.2)			
Education	Quran/Madrasah	78(74)	861(88)	18.4	1	0.000
	Primary	28(26)	113(11)			

**Table-III:** Anthropometry of the households.

*Anthropometry of the households*						

*Variables*		*Diabetes n=106 (%)*	*No diabetes n =974 (%)*	*X^2^*	*df*	*p-value*
BMI	Under weight	3(3)	79(8.1)	44	3	0.000
	Normal	23(22)	454(46)			
	Overweight	35(33)	260(27)			
	Obese	45(42)	181(19)			
Waist Hip	Ratio					
	Normal	10(9.4)	230(24)	44	3	0.000
	Central obesity	96(91)	744(76)			

**Table-IV:** Behavioral pattern and health advice.

		*Behavioral pattern and health advice among the household*				

*Variables*		*Diabetes n=106(%)*	*No diabetes n =974(%)*	*X^2^*	*df*	*p-value*
Family history of diabetes	Yes	47(44)	59(6)	47	1	0.000
	No	59(56)	915(94)			
Drug for control of diabetes	Yes	61(58)	45(5)	473	1	0.000
	No	45(42)	929(95)			
Advice to do exercise	Yes	48(45)	58(6)	111	1	0.000
	No	58(55)	916(94)			
Advice to reduce weight	Yes	48(45)	58(6)	135	1	0.000
	No	58(55)	916(94)			
Advice to stop smoking	Yes	14(13)	92(9)	32	1	0.000
	No	92(87)	882(91)			
Advice to take special diet	Yes	56(53)	50(5)	149	1	0.000
	No	50(47)	924(95)			

Declaration of interest: None. Funding: None.

## DISCUSSION

The global burden of diabetes is showing a rapid rise over the past few decades. The rapid urbanization, changing life style lack of physical activity and eating habits are all contributing factors.^2^ In the current study the calculated prevalence of diabetes among the households was found to be 9.8% which when compared with the prevalence quoted by National Diabetic survey^7^ was 11.1% much less and also much less than the prevalence cited by study in the conducted in Union councils of Rawalpindi which was 32.9%.^8^ The National Urban Survey conducted across metropolitan cities of India showed a prevalence from 6.1% to 16.6%.^9^ The current state of diabetes in Bangladesh is also showing a rising trend with reported numbers of 7.4% which is lesser than our figure.^10^ The reason might be that, the statistics shown by the current study is just a snapshot as the data was collected from a particular targeted community. The number could have been more if the data would have collected from a larger and scattered community.

As obesity is considered to be an independent risk factor of Type-II diabetes^9^,^11^ the same had been depicted by our results. In present study the measures of obesity that is BMI and WHR have been found to be significantly associated with diabetes. The same is shown by study in which markers of obesity had also shown the same results which highlighted that increasing BMI and diabetes are interrelated.^11^ The possible reason could be that local cortisol metabolism in adipose tissue in obese individuals leads to poor glycemic control and hence insulin resistance.^11^ It is worth mentioning to emphasize here that among the anthropometric measures WHR is better predictor of obesity which can lead to cardiovascular comorbidities and help us determine the visceral fat distribution.^12^ So clinician taking care of theses diabetic patient should counsel them about the threatening possibilities which can arise in them due to increasing BMI and central obesity.

The rising trend of modern epidemic like diabetes is more affecting the urban population than the rural.^4^,^10^,^13^ Every developing country, including the subcontinent is showing the same inclination.^9^,^13^ In the current study the data had been collected from the periurban community, statistic of diabetes in this population is suggestive that life style changes and unhealthy eating habits were the biggest reasons which has led to obesity and deranged glycemic controls.^5^ The data from different studies from around the world is also showing the same pattern. Moreover, positive family history had also amplified the picture.^13^

## CONCLUSION

The diabetes is significantly associated with positive family a history and deranged BMI both overweight and obesity along with central obesity. This can only be prevented by health education and life style modifications.

### Authors’ contributions

**HA:** Conceptualization of main concept of this project, Literature search, data collection, data compilation and analysis and development of the discussion and conclusion.

**IT:** Conceptualization of main concept of this project, data analysis, writing of the discussion and conclusion.

**IM:** Data analysis, writing of the discussion and conclusion.
